# Factors associated with initiation and exclusive breastfeeding at hospital discharge: late preterm compared to 37 week gestation mother and infant cohort

**DOI:** 10.1186/1746-4358-7-16

**Published:** 2012-11-26

**Authors:** Jennifer Ayton, Emily Hansen, Stephen Quinn, Mark Nelson

**Affiliations:** 1 School of Sociology and Social Work, University of Tasmania, Hobart, Australia; 2 Menzies Research Institute Tasmania, University of Tasmania, Hobart, Australia; 3 Flinders Clinical Effectiveness, Flinders University, Adelaide, South Australia, Australia

**Keywords:** Exclusive breastfeeding, Initiation, Late preterm, Infant

## Abstract

**Background:**

To investigate and examine the factors associated with initiation of, and exclusive breastfeeding at hospital discharge of, late preterm (34 ^0/7^ - 36 ^6/7^ weeks) compared to 37 week gestation (37 ^0/7^ - 37 ^6/7^ week) mother and baby pairs.

**Methods:**

A retrospective population-based cohort study using a Perinatal National Minimum Data Set and clinical medical records review, at the Royal Hobart Hospital, Tasmania, Australia in 2006.

**Results:**

Late preterm and 37 week gestation infants had low rates of initiation of breastfeeding within one hour of birth, 31 (21.1%) and 61 (41.5%) respectively. After multiple regression analysis, late preterm infants were less likely to initiate breastfeeding within one hour of birth (OR 0.3 95% CI 0.1, 0.7 p = 0.009) and were less likely to be discharged exclusively breastfeeding from hospital (OR 0.4 95% CI 0.1, 1.0 p = 0.04) compared to 37 week gestation infants.

**Conclusion:**

A late preterm birth is predictive of breastfeeding failure, with late preterm infants at greater risk of not initiating breastfeeding and/or exclusively breastfeeding at hospital discharge, compared with those infants born at 37 weeks gestation. Stratifying breastfeeding outcomes by gestational age groups may help to identify those sub-populations at greatest risk of premature cessation of breastfeeding.

## Background

Late preterm infants (LPI), loosely classified by current literature as infants born 34 ^0/7^ - 36 ^6/7^ weeks gestation (239 days through to 259 days) [1-3], are less studied and described within Australian literature than term infants. Consensus does not exist around the gestational age group classification for this sub group of preterm infants, often cited as moderately preterm, near term, mildly preterm with gestational age ranges between 32 through to 37 weeks.

Late preterm births have contributed disproportionally to the rising incidence of preterm births within the last decade [4,5], adding substantially to the overall impact on health care services, both in the acute, and within primary health care settings [4,6-8]. In Australia they make up 69% of all preterm births and 8.1% of all births [8] and are five times more common than births occurring before 32 weeks gestation [9]. Compared with infants born at, and within, the term gestational period (37 ^0/7^ weeks (260 days) through to 41^6/7^ weeks (294 days) [10], late preterm infants are at increased risk of neonatal morbidity, experiencing one or more short term and long term health outcomes (hypoglycaemia, hypothermia, jaundice, delayed oral feeding, readmission to hospital, transient tachypnea [1,11], neuro-developmental delays [12] and mortality) [13,14]. Despite the importance of breastfeeding for this vulnerable population of infants, the breastfeeding outcomes of preterm infants are less well documented and monitored compared to term infant populations [15].

The World Health Organization (WHO) recommends early initiation (where the infant receives colostrum or is breastfeed within the first hour of birth) and exclusive breastfeeding up to 6 months for all infants [16]. Exclusive breastfeeding is the gold standard of infant nutrition, defined as the infant was fed breast milk only (including expressed breast milk, oral rehydration solutions, drops, syrups, vitamins, minerals, medicines) [17].

Those studies that have reported outcomes by gestational age have found that infants born at 35–36 weeks gestation are less likely to initiate breastfeeding, compared to infants born at 37–39 weeks gestation, (88.2% compared to 92.0% respectively) [18]. Those infants born 35–36 weeks gestation had a lower incidence of breastfeeding at 6 months compared to those born ≥ 37–39 weeks [18]. In the Pelotas birth cohort study, late-preterm infants were 10% more likely not to commence breastfeeding or receive breast milk within the first 24 hours of life than term infants [19].

Many factors are negatively associated with successful breastfeeding, resulting in delays in, and/or failure of, early breastfeeding initiation (within the first hour of birth) and reduced duration of exclusive breastfeeding for term infant populations including; mode of delivery (caesarean versus vaginal birth), mothers parity (primparous women), maternal smoking, insufficient milk supply, maternal obesity [20,21]. It is therefore important to identify potential modifiable factors that contribute to breastfeeding failure (initiation and exclusive) for late preterm infants in order to customise breastfeeding support strategies that can address these factors in the clinical setting.

The aim of this study is to describe and investigate the breastfeeding outcomes of a mother and infant cohort of a sub population of preterm infants, LPI (34 ^0/7^ - 36 ^6/7^ weeks) compared to a 37 week gestation (37 ^0/7^ - 37 ^6/7^) cohort and to identify the infant and maternal factors associated with initiation and exclusive breastfeeding at discharge amongst both cohorts.

## Methods

### Design

This was a retrospective population-based cohort study using a Perinatal National Minimum Data Set and hospital clinical medical records review.

### Setting and sample

The study was conducted at the Royal Hobart Hospital (RHH), Tasmania, Australia, a 501-bed WHO/UNICEF Baby Friendly accredited facility and Tasmania’s major teaching and tertiary referral centre for high and low risk obstetrics. The sample was purposefully selected from the larger Royal Hobart 2006 birth population systematically. Data on mother and infant pairs who had given birth within the calendar year of 2006 were obtained from the Tasmanian Perinatal National Minimum Data Set [22]. The Tasmanian perinatal data set is derived from the Australian National Perinatal Data Collection, which is core set of data elements endorsed by the National Health Information Standards and Statistics Committee for mandatory collection and reporting of pregnancy and childbirth outcomes of mothers and babies.

### Inclusion criteria

Live births (in 2006) born between 34 ^0/7^ through to 37 ^6/7^ weeks gestation (239–266 days since the first day of the mothers last menstrual period), surviving to hospital discharge and whose mothers had intended to breastfeed in the antenatal period.

### Exclusion criteria

Infants with congenital abnormities, infants transferred in or out of the facility, and mothers who elected not to breastfeed were excluded.

### Definitions and terms used

Infant gestational age was estimated by obstetric ultrasound and/or calculated using the first day of the mother’s last normal menstrual period, documented in the mother’s antenatal clinical medical records and verified against the Tasmanian perinatal data set.

Preterm birth defined as those infants born less than 37 completed weeks of gestation (28 ^0/7^ weeks through to (196 days) 36 ^6/7^ weeks gestation (259 days) from the mothers last menstrual period) [10]. Term birth defined as infants born from 37 ^0/7^ weeks (260 days) through to 41^6/7^ weeks (294 days) from the mother’s last menstrual period [10]. For the purposes of this study the 37 week gestation cohort included infants born 37 ^0/7^ weeks (260 days) through to 37 ^6/7^ weeks gestation (266 days).

### Breastfeeding terms

The terms used to measure the breastfeeding outcomes for this study are derived from the WHO recommended definitions [17]. ‘Exclusive breastfeeding’ is defined as the infant was fed breast milk only (including expressed breast milk, oral rehydration solutions, drops, syrups, vitamins, minerals, medicines). ‘Initiation of breastfeeding’ defined as the infant breastfed or received colostrum within one hour of birth [17]. Mothers’ ‘intention to breastfeed’ was obtained from duplicate baby and mother clinical perinatal birth records and verified against the mothers’ antenatal clinical medical records.

### Data collection

Mother and infant demographic variables, method of birth, Apgar scores, birth weight (grams), gestational age, parity (i.e. number of previous pregnancies excluding the current pregnancy), postcode, date of birth, discharge date/time electronically extracted from the Tasmanian Perinatal National Minimum Data Set, cross-checked twice against mother and infant medical records for validity and entered into an Microsoft Access data base. Breastfeeding practices (initiation of, and exclusive breastfeeding as per WHO recommended definitions described above) [17], were extracted from the maternal and infant clinical records and verified against the mother and infant discharge summary. Ethics approval was granted from Southern Tasmania Health and Medical Human Research Ethics Committee [H0009470].

### Data analysis

Infant gestational age was dichotomised into 37 week group (37 ^0/7^ - 37 ^6/7^ week gestation) and late preterm (34 ^0/7^ - 36 ^6/7^ weeks gestation) and sample characteristics compared using *t-*tests or chi-squared tests as appropriate*.* Multivariable logistic regression was used to examine the associations between outcomes, initiation of breastfeeding and exclusive breastfeeding, and our predictor of interest preterm / 37 week gestation infants. These models were adjusted for potential confounding factors listed as footnotes in each table. All models were validated using the Hosmer-Lemeshow goodness of fit statistic. A p-value of 0.05 (two-tailed) is considered statistically significant. All statistical analysis was conducted using Stata version 10.

## Results

Maternal and infant characteristics are summarized in Table 1. During this study period (2006) there were 1887 live births at the RHH, 1730 (92%) were born between 37 ^0/7^ - 42 ^6/7^ weeks gestation and 156 (8%) were born preterm gestation (28 ^0/6^ - 36 ^6/7^ weeks).

**Table 1:** Maternal and infant characteristics; late preterm compared with 37 week gestation, infant and mother cohort (Values are in n (%) or mean ± SD)

**Variable**	**37 weeks**	**34-36 weeks**	**p value**
**Mean ± SD**	**n = 80**	**n = 67**	
Maternal			
Parity	1 ± 1.3	1 ± 1.3	0.48
Maternal smoking	28 (38.9)	23 (38.3)	0.95
Maternal age	28.3 ± 6.8	27.3 ± 5.9	0.30
Infant			
^†^Caesarean birth	47 (57.5)	41 (61.2)	0.56
Birth weight (grams)	2944 ± 462.3	2556 ± 488.7	<0.001
Apgar at one minute	8 ± 1.5	8 ± 1.7	0.08
Multiple Birth	6 (7.5)	4 (6.0)	0.71
^††^Initiated breastfeeding	61 (41.5)	31 (21.1)	<0.001
^†††^Exclusive breastfeeding at discharge	67 (83.7)	40 (59.7)	<0.001

^†^Caesarean birth combined data (elective and emergency lower uterine segment caesarean section).

^† †^Initiated breastfeeding – breastfed or received colostrum within one hour of birth.

^†††^Exclusive breastfeeding at discharge, within 24 hours of discharge.

Infants born late preterm, 34 ^0/6^ - 36 ^6/7^ weeks gestation represented 108 (60%) of all preterm births within the study period. A total of n = 147 (67 (46%) late preterm and 80 (54%) 37 week gestation infants) were eligible for inclusion in this study based on maternal intent to breastfeed, the infant was born between 34 ^0/7^ through to 37 ^6/7^ weeks gestation and survived to hospital discharge. Of these, 41 (61%) late preterm births were born via lower uterine caesarean section (elective and emergency combined) (Table 1).

Initiation of breastfeeding within one hour of birth was significantly lower for late preterm infants 31 (21%) when compared to 37 week gestation infants 61 (41%) (p = <0.001) (Table 1). Late preterm infants born by lower uterine caesarean section were 80% less likely initiate breastfeeding within one hour of birth (p = <0.002) (Table 2).

**Table 2:** **Factors associated with **^**††**^** initiation of breastfeeding; late preterm compared with 37 week gestation, infant and mother cohort**

**Variable**	**Univariable OR (95% CI)**	**p value**	*Multivariable OR (95% CI)	**p value**
Preterm (34-36wk)	0.3 (0.1, 0.5)	<0.001	0.3 (0.1, 0.7)	0.009
Parity	0.8 (0.6, 1.0)	0.15	1.7 (0.4, 1.2)	0.21
Maternal smoking	1.1 (0.5, 2.3)	0.73	1.1 (0.4, 2.8)	0.79
Maternal age	1.0 (1.0, 1.1)	0.538	1.1 (1.0, 1.1)	0.05
Birth weight, g	1.0 (1.0, 1.0)	0.08	1.0 (1.0, 1.0)	0.72
Apgar at one minute	1.6 (1.3, 2.0)	<0.001	1.7 (1.2, 2.2)	<0.001
^†^Caesarean birth	0.3 (0.1, 0.7)	0.004	0.2 (0.1, 0.6)	<0.002
Multiple birth	0.86 (0.21, 3.62)	0.84	0.5 (0.1, 2.8)	0.41

*Adjusted for maternal smoking, age, parity, infant birth weight, caesarean birth, multiple birth, Apgar at one minute.

^†^Caesarean section, combined data, emergency and elective caesarean births.

^††^ Initiated breastfeeding, infant breastfed or received colostrum within one hour of birth.

Tables 2 and 3 show the univariable and multivariable model obtained by logistic regression for the factors associated with initiation of breastfeeding within one hour of birth and exclusive breastfeeding at discharge. Late-preterm infants were 70% less likely to initiate breastfeeding, and 60% less likely to be discharged exclusively breastfeeding from hospital when compared to 37 week gestation infants (Table 3).

**Table 3:** Factors associated with exclusive breastfeeding at discharge; late preterm compared with 37 week gestation, infant and mother cohort

**Variable**	**Univariable OR (95% CI)**	**p value**	*Multivariable OR (95% CI)	**p value**
Preterm (34-36wk)	0.3 (0.1, 0.6)	<0.001	0.4 (0.1, 1.0)	0.04
Parity	1.4 (1.0, 2.0)	0.05	1.3 (0.9, 1.9)	0.15
Maternal smoking	1.2 (0.5, 2.7)	0.61	1.2 (0.5, 3.1)	0.62
Maternal age	1.0 (1.0, 1.1)	0.22	1.0 (0.9, 1.1)	0.62
Birth weight, g	1.0 (1.0, 1.0)	0.01	1.0 (0.9, 1.0)	0.11
^†^Caesarean birth	0.7 (0.3, 1.6)	0.48	0.4 (0.2, 1.1)	0.09
Multiple birth	0.9 (0.2, 3.5)	0.84	1.2 (0.2, 6.3)	0.79

*Adjusted for maternal smoking, age, parity, caesarean birth, infant birth weight, multiple births.

^†^Caesarean section, combined data emergency and elective caesarean birth.

## Discussion

To our knowledge, this is the first time that the breastfeeding practices (initiation of and exclusive breastfeeding) of late preterm infants (34 ^0/7^ - 36 ^6/7^ weeks) have been reported in the Australian context. The proportion of late preterm infants to commence breastfeeding was much lower than those reported for infants born at similar gestations in other international studies [18,19]. Late preterm infants were significantly less likely to initiate breastfeeding within one hour of birth, or to be discharged home exclusively breastfeeding, compared to infants in the 37 weeks group. In this study, an important factor for predicting breastfeeding failure was gestational age and a cesarean section birth, with those infants born between 34 ^0/7^ - 36 ^6/7^ weeks gestation at highest risk of failure. Gestational age, namely a late preterm birth, is predictive of breastfeeding failure.

An unexpected finding from this study was the high proportion of late preterm infants delivered by a lower uterine cesarean section (elective and emergency combined). In Australia, 30.8% of women gave birth via caesarean section in 2006, increasing to 35.5% in 2009 [23,24]. Similar upward trends have been observed and reported in larger studies for other high income countries with the rising incidence of late preterm births being attributed to increasing obstetric intervention, namely caesarean section [4,5]. Late preterm infants delivered by caesarean section in this study were 80% less likely to initiate breastfeeding within one hour of birth (p = < 0.002). Larger studies amongst term infant/mother populations have suggested negative associations between a caesarean delivery (elective and emergency) and onset of lactation and milk transfer and volume produced [21,25]. Further, Cregan et al. found that 82% of women delivering prematurely experienced problems with initiation, and had significant reduction in the volume of milk produced [26]. The combination of a caesarean delivery and prematurity of the infant creates a complex feeding scenario. Compromised by the varying degrees of infant physiological and developmental maturity reported in other studies [5,11,27], the late preterm infant and mother experience a cascade of events, such as ineffective sucking at the breast leading to poor intake of milk volume, delayed and low production of maternal milk, infant lethargy, sleepiness, hypoglycaemia and hypothermia. Efforts should be made to review clinical care policies and reduce the number of caesarean section deliveries so as to improve the late preterm mother and infant chances of initiating and maintaining exclusive breastfeeding.

In population terms, late preterm births represent the largest subset of preterm births born at this facility and nationally [8]. The size of this population alone is likely to impact on health outcomes and healthcare resources [1,11,12,19,28]. Indeed, it is worth considering what impact preterm infant populations have on national breastfeeding rates, where they are not currently reported separately [29]. Stratifying breastfeeding outcomes by gestational age may help to identify those sub-populations at greatest risk of premature cessation.

Late preterm infants present with subtle problems that predispose them to poor breastfeeding outcomes. Notably, they are less likely to initiate at birth and to be discharged exclusively breastfeeding. The combined complexity of a caesarean delivery, infant prematurity and breastfeeding exacerbates the potential for problems for late preterm mothers and infants. A simple reminder for clinicians is; as gestational age decreases so does the chances of breastfeeding success.

The purpose of this article is to identify issues and areas for intervention, increase awareness and prompt a review of care policy and practices at the local and national levels. The data presented supports the current and accumulating evidence suggesting that infants born 34 ^0/7^ - 36 ^6/7^ weeks gestation represent the largest preterm infant birth population and are at increased risk of adverse clinical outcomes and breastfeeding problems while in hospital and indeed potentially after discharge. The low proportion of late preterm infants successfully commencing breastfeeding within the first hour after birth and then being discharge home exclusively breastfeeding attests to the vulnerability of this population and a growing body of evidence pointing to the increased morbidity of these infants.

### Limitations

The data for this study were taken from a single centre, thus providing a small study number and impacting on the generalisability of the findings. Data extraction and analysis was dependent on the quality of primary data sources from the Tasmanian perinatal database and clinical medical notes.

It was outside the scope of this study to compare late preterm to other term gestation infant groups such as 37 ^0/7^ through to 39 ^6/7^ weeks gestation. We recognized that 37 week gestation (now called ‘early term’) infants may still not have optimal breastfeeding initiation and exclusive breastfeeding rates when compared with infants born greater or equal to 39 weeks gestation.

## Conclusions

Gestational age, namely a late preterm birth, is predictive of breastfeeding failure.

Late preterm infants are at greater risk of not initiating breastfeeding or exclusively breastfeeding at hospital discharge when compared to 37 week gestation infants. Gestational age and a caesarean delivery are modifiable risk factors negatively effecting breastfeeding success for mothers and infants. Infants born 34 ^0/7^ - 36 ^6/7^ weeks gestation represent the largest preterm infant birth population within Australia. The size of this preterm birth sub-group alone warrants further investigation and closer monitoring, particularly in relation to breastfeeding outcomes. Stratifying breastfeeding outcomes by gestational age groups may help to identify those sub-populations at greatest risk of premature cessation.

The data presented in this study act as a necessary first step for further research exploring reasons for breastfeeding failure and what strategies are needed to support and facilitate successful and continued exclusive breastfeeding for late preterm infants and their mothers.

## Competing interests

The authors declare that they have no competing interests.

## Authors’ contributions

JA carried out the conception and design and acquisition of all the data and support with the analysis and interpretation of the data, preparation and writing of the manuscript. SQ carried out data analysis, supported with the interpretation of the data and revision of the manuscript and prepared the statistical analysis section in the manuscript. EH and MN provided support with conception design and interpretation of the data and detailed revision of manuscript. All authors read and approved the final manuscript.
